# Records of medical malpractice litigation: a potential indicator of health-care quality in China

**DOI:** 10.2471/BLT.16.179143

**Published:** 2017-03-13

**Authors:** Zhan Wang, Niying Li, Mengsi Jiang, Keith Dear, Chee-Ruey Hsieh

**Affiliations:** a Duke Kunshan University, No 8 Duke Avenue, Kunshan, Jiangsu Province, 215316, China.

## Abstract

**Objective:**

To assess the characteristics and incidence of medical litigation in China and the potential usefulness of the records of such litigation as an indicator of health-care quality.

**Methods:**

We investigated 13 620 cases of medical malpractice litigation that ended between 2010 and 2015 and were reported to China’s Supreme Court. We categorized each case according to location of the court, the year the litigation ended, the medical specialization involved, the severity of the reported injury, the type of allegation raised by the plaintiff – including any alleged shortcomings in the health care received – and the outcome of the litigation.

**Findings:**

The annual incidence of medical malpractice litigation increased from 75 in 2010 to 6947 in 2014. Most cases related to general surgery (1350 litigations), internal medicine (3500 litigations), obstetrics and gynaecology (1251 litigations) and orthopaedics (1283 litigations). Most of the reported injuries were either minor (1358 injuries) or fatal (4111 deaths). The most frequent allegation was of lack of consent or notification (1356 litigations), followed by misdiagnosis (1172 litigations), delay in treatment (1145 litigations) and alteration or forgery of medical records (975 litigations). Of the 11 014 plaintiffs with known litigation outcomes, 7482 (67.9%) received monetary compensation.

**Conclusion:**

Over our study period, the incidence of litigation over potential medical malpractice increased in China. As many of the cases related to alleged inadequacies in the quality of health care, records of medical malpractice litigation in China may be worth exploring as an indicator of health-care quality.

## Résumé

**Objectif:**

Évaluer les caractéristiques et l'incidence des litiges médicaux en Chine et l'utilité potentielle des données disponibles sur ces litiges en tant qu'indicateurs de la qualité des soins de santé.

**Méthodes:**

Nous avons étudié 13 620 actions en justice liées à de mauvaises pratiques médicales, qui se sont terminées entre 2010 et 2015 et ont été portées à la connaissance de la Cour suprême de Chine. Nous avons classé chaque action en justice en fonction de l'emplacement du tribunal saisi, de l'année de résolution du litige, de la spécialité médicale impliquée, de la gravité du préjudice signalé, du type d'allégation de la partie demanderesse (y compris des insuffisances dans les soins dispensés) et de l'issue du litige.

**Résultats:**

L'incidence annuelle des actions en justice liées à de mauvaises pratiques médicales est passée de 75 cas en 2010 à 6 947 en 2014. La plupart de ces affaires étaient liées à la spécialité de chirurgie générale (1 350 litiges), à la médecine interne (3 500 litiges), à la médecine obstétrique et gynécologique (1 251 litiges) et à la médecine orthopédique (1 283 litiges). Dans la majorité des cas, les préjudices signalés ont été mineurs (1 358 blessures mineures) ou fatals (4 111 décès). L'allégation la plus souvent avancée a été une absence de consentement ou d'information (1 356 cas), suivie par une erreur de diagnostic (1 172 cas), un retard dans la mise en place du traitement (1 145 cas) et l'altération ou la falsification des dossiers médicaux (975 cas). Sur les 11 014 parties demanderesses impliquées dans les actions en justice dont nous connaissons l'issue, 7 482 (67,9%) ont perçu une indemnisation financière.

**Conclusion:**

Sur la période que nous avons étudiée, l'incidence des actions en justice liées à de potentielles mauvaises pratiques médicales a augmenté en Chine. Étant donné que de nombreuses affaires portent sur des inadéquations potentielles de la qualité des soins de santé, les données disponibles sur les actions en justice liées aux mauvaises pratiques médicales en Chine pourraient éventuellement être exploitées en tant qu'indicateurs de la qualité des soins de santé.

## Resumen

**Objetivo:**

Evaluar las características y la incidencia de litigios médicos en China y la posible utilidad de los registros de dichos litigios como un indicador de la calidad de la atención sanitaria.

**Métodos:**

Se investigaron 13 620 casos de litigios por negligencias médicas que acabaron entre 2010 y 2015 y se enviaron al Tribunal Supremo de China. Cada caso se categorizó según la ubicación del tribunal, el año de finalización del litigio, la especialidad médica implicada, la gravedad del daño denunciado, el tipo de denuncia planteada por el demandante (incluida cualquier deficiencia de la atención sanitaria recibida) y el resultado del litigio.

**Resultados:**

La incidencia anual de los litigios por negligencias médicas aumentó de 75 en 2010 a 6 947 en 2014. La mayoría de los casos estaban relacionados con cirugía general (1 350 litigios), medicina interna (3 500 litigios), obstetricia y ginecología (1 251 litigios) y ortopedia (1 283 litigios). La mayoría de los daños denunciados fueron o menores (1 358 daños) o mortales (4 111 mortales). La denuncia más frecuente fue la ausencia de consentimiento o notificación (1 356 litigios), seguida del diagnóstico equivocado (1 172 litigios), el retraso del tratamiento (1 145 litigios) y la alteración o falsificación de registros médicos (975 litigios). De los 11 014 demandantes con resultados de litigación conocidos, 7 482 (67,9%) recibieron una compensación económica.

**Conclusión:**

Durante el periodo de estudio, la incidencia de los litigios por posibles negligencias médicas aumentó en China. Dado que muchos de los casos estaban relacionados con la denuncia de deficiencias en la calidad de la atención sanitaria, puede ser necesaria una exploración de los registros de litigios por negligencias médicas en China a modo de indicador de la calidad de la atención sanitaria.

## ملخص

**الغرض:**

تقييم خصائص الدعاوى القضائية للحالات الطبية في الصين ومدى تأثيرها بالإضافة إلى الفوائد المحتملة لسجلات مثل تلك الدعاوى القضائية كمؤشر لجودة الرعاية الصحية.

**الطريقة:**

قمنا بالتحري عن 13,620 حالة من حالات الدعاوى القضائية المتعلقة بالإهمال الطبي التي انتهت في الفترة بين 2010 و2015 وتم إبلاغ المحكمة العليا الصينية بشأنها. كما قمنا بتصنيف كل حالة وفقًا لموقع المحكمة، وتاريخ انتهاء الدعوى القضائية، والتخصص الطبي المعني، وشدة الإصابة المبلغ عنها، وموضوع الدعوى الذي أثاره المدعي – بما في ذلك أي قصور مزعوم في الرعاية الصحية المقدمة – ونتيجة الدعوى القضائية.

**النتائج:**

ارتفع المعدل السنوي للدعاوى القضائية المتعلقة بالإهمال الطبي من 75 حالة في عام 2010 إلى 6947 في عام 2014. وكانت أغلب الحالات تتعلق بالجراحة العامة (1350 دعوى قضائية)، والطب الباطني (3500 دعوى قضائية)، وأمراض النساء والتوليد (1251 دعوى قضائية) وطب العظام (1283 دعوى قضائية). كما كانت أغلب الإصابات المبلغ عنها إما طفيفة (1358 إصابة) أو قاتلة (4111 حالة وفاة). وكانت الدعاوى القضائية الأكثر شيوعًا تتعلق بعدم موافقة المريض أو إبلاغه (1356 دعوى قضائية)، يتبعها التشخيص الخاطئ (1172 دعوى قضائية)، والتأخير في العلاج (1145 دعوى قضائية)، وتغيير السجلات الطبية أو تزويرها (975 دعوى قضائية). من بين 11,014 مدعيًا من أصحاب الدعاوى القضائية المفصول فيها، حصل 7482 (67.9‏%) منهم على تعويض مالي.

**الاستنتاج:**

على مدى فترة الدراسة، ارتفع معدل الدعاوى القضائية في الصين فيما يتعلق بالإهمال الطبي وارد الحدوث. ونظرًا لأن العديد من الحالات تتعلق بأوجه القصور المزعومة في جودة الرعاية الصحية، فقد تكون سجلات الدعاوى القضائية المتعلقة بالإهمال الطبي في الصين جديرة بالاستكشاف باعتبارها مؤشرًا على جودة الرعاية الصحية.

## 摘要

**目的：:**

旨在评估中国医疗诉讼的特征和发生率以及使用此类诉讼的记录作为医疗护理质量指标的潜在作用。

**方法：:**

我们调查了在 2010 至 2015 年间结案并呈报给中国最高法院的 13 620 件医疗事故诉讼案例。 我们根据法院所在地、诉讼结案年份、涉及的医疗规范、呈报损害的严重程度、原告提起的指控类型（包括接受的医疗护理中被指控的任何不足）以及诉讼结果对各案件进行了分类。

**结果：:**

医疗事故诉讼的年发生率由 2010 的 75 例增加到 2014 年的 6947 例。大多数案例与普通外科（1350 例）、内科（3500 例）、产科及妇科（1251 例）以及骨科（1283 例）有关。 大多数呈报损害的程度为轻微（1358 件）或致命（4111 件）。 最常见的指控是未征得同意或通知（1356 例），其次是误诊（1172 例）、延误治疗（1145 例）以及医疗记录变更或造假（975 例）。 在已知诉讼结果的 11 014 名原告当中，7482 (67.9%) 人获得了金钱赔偿。

**结论：:**

在研究期间，中国潜在医疗事故诉讼的发生率有所增加。 由于许多案例均与指控医疗护理质量不足有关，因此，将中国的医疗事故诉讼记录作为一项医疗护理质量指标进行探究可能很有价值。

## Резюме

**Цель:**

Дать оценку характерным особенностям и частоте инициирования судебных разбирательств по медицинским делам в Китае и потенциальной применимости записей о таких судебных разбирательствах в качестве показателя качества медико-санитарного обслуживания.

**Методы:**

Авторы изучили 13 620 случаев судебных разбирательств по делам о недобросовестной медицинской практике, которые завершились в период между 2010 г. и 2015 г. и о которых было сообщено в Верховный суд Китая. Каждый случай был классифицирован в зависимости от местоположения суда, года завершения судебного разбирательства, вовлеченной медицинской специальности, степени тяжести заявленной травмы, типа выдвинутых истцом обвинений, включающих какие-либо обвинения в неполноценности оказываемых медико-санитарных услуг, и результата судебного разбирательства.

**Результаты:**

Годовая частота инициирования судебных разбирательств по делам о недобросовестной медицинской практике увеличилась с 75 разбирательств в 2010 году до 6947 в 2014 году. Большинство случаев было связано с разделом общей хирургии (1350 разбирательств), внутренних болезней (3500 разбирательств), акушерства и гинекологии (1251 разбирательство) и ортопедии (1283 разбирательства). Большинство заявленных травм были легкой степени тяжести (1358 травм) или смертельными (4111 смертей). Наиболее часто выдвигались обвинения в отсутствии согласия или официального уведомления (1356 разбирательств), постановке неправильного диагноза (1172 разбирательства), несвоевременном предоставлении лечения (1145 разбирательств) и изменении или подделке медицинских записей (975 разбирательств). Из 11 014 истцов, участвовавших в судебных разбирательствах с известными результатами, 7482 (67,9%) получили денежную компенсацию.

**Вывод:**

В течение исследуемого периода частота инициирования судебных разбирательств по делам о возможной недобросовестной медицинской практике в Китае возросла. Поскольку многие судебные разбирательства были связаны с вменяемой в вину неудовлетворительностью медико-санитарного обслуживания, может быть целесообразным исследовать возможность применения записей о судебных разбирательствах по делам о недобросовестной медицинской практике в Китае в качестве показателя качества медицинской помощи.

## Introduction

The quality of health care can be difficult to measure,^1^ but many potentially relevant indicators have been investigated. In high-income countries, case report cards are frequently and widely used to record health outcomes – including adverse outcomes that may sometimes be attributable to poor health care – and health providers may also employ quality assurance systems such as error-reporting mechanisms. In many low- and middle-income countries, however, the lack of such records and systems is a major obstacle to measuring the quality of health care.^2^

In theory, patients should be protected from some aspects of poor health care by the litigation relating to medical malpractice. Such litigation can lead to financial compensation for patients who have suffered as the result of medical negligence and can encourage physicians to maintain at least basic standards of health care.^1^ In high-income countries, such as the United States of America (USA), researchers have examined the quality of health care from the perspective of medical errors and have concluded that – even though “to err is human” – most errors in the provision of health care could be prevented.^3^ However, as an effective approach to prevent medical errors in particular and to increase the quality of health care in general, the operation of the medical malpractice system in the USA is far from ideal.^4^

Litigation records have rarely been used as an external data source for studies on medical malpractice.^5^^–^^8^ However, in the investigation of health-care quality, a potential strength of using such records is that health providers may lack the incentive to report comprehensively and honestly unless they are in court and being accused of negligence. Litigation records may also allow the quality of care to be assessed from the perspective of both patient and provider. Although such records may cover only a very small proportion of all adverse outcomes,^9^^,^^10^ they can still be useful indicators of the general quality of health care in a country. When, for example, patient injury claims from Finnish hospital registries for 1998–2003 were investigated, some more traditional indicators of poor hospital care – such as high prevalences of infection – were found to be significantly associated with the incidence of claims and compensation.^11^

We need a better understanding of the relationship between adverse health outcomes, medical malpractice claims and poor health care. Donabedian divided the measurement of health-care quality into three categories: (i) outcomes, that is the health status of patients after they had received care; (ii) process, i.e. the procedures involved in diagnosis and treatment; and (iii) structure, i.e. the human and material resources available and the infrastructure of the health system.^12^ The relationship between the structural components of quality and patient outcomes has been investigated.^13^^,^^14^ Not all adverse health outcomes can be attributed to health care of poor quality. In addition, attempts to improve the quality of health-care services may not always result in improved health outcomes.^15^ For example, if litigation is increased to reduce medical negligence, physicians may become so fearful of being sued that they turn towards so-called defensive medicine^4^ – which may well be suboptimal in terms of the health outcomes of the physicians’ patients.

It should be possible to use litigation records, from a fault-based legal system, to identify the court cases where adverse outcomes were not attributed to the poor quality of care, i.e. where there was litigation but no consequent compensation. Such records are unlikely to reveal any medical negligence that did not adversely affect the health outcomes of the patients involved. They also give no indication of the numbers of patients who, although they have suffered adverse consequences of medical negligence, chose not to seek any compensation in a court of law.

In China, although public hospitals provide approximately 90% of health-care services, health care from the private sector has grown rapidly since 2009, when national reform of health care promoted universal health coverage.^16^ Over the same period, there appears to have been a deterioration in the general relationship between patients and providers and an increase in medical litigation and violence against health professionals.^17^^–^^21^ There is a need to resolve such issues but data on health-care quality and useful indicators of such quality are rare in China.^22^^,^^23^ Some hospitals are investing in systems based on electronic medical records but such systems are not well integrated and the recorded data are rarely shared between hospitals or assimilated at regional level.^24^

Some shortcomings in the quality of health care in China were revealed when 1086 records of medical malpractice litigation, from a third-party database, were investigated – but the sample selection method employed to create the database could not be determined.^5^ Of the 504 physicians recorded in a cross-sectional survey in the Chinese city of Shenzhen, only 10 (2.0%) reported that they had been involved in medical malpractice litigation.^25^ Despite the apparent rarity of such litigation, we wondered if, in China, the analysis of records of medical malpractice might provide a useful – and previously under-researched – method of assessing health-care quality and the temporal trends in such quality. We therefore collected and investigated the publicly available records, from the Supreme Court of China, of cases of medical malpractice litigation that ended between 2010 and 2015.

## Methods

### Setting

In China, when a negligence-related adverse outcome occurs, the patient or the patient’s family members may negotiate directly with the health-care provider – over liability and compensation – or they may file a lawsuit. For the lawsuits, which usually end in a civil judgement or civil ruling, all the related information on the plaintiff and defendant, any medication involved, the adverse outcomes, the evidence of potential negligence, the legal debates, the relevant legislation and case outcome should be recorded, submitted to the Supreme Court of China and then made available publicly via an electronic database called China Judgments Online.^26^ By November 2016, this database covered over 20 million litigation records and had had over 3.9 billion visits.^26^

### Selection of records

**Fig. 1:**
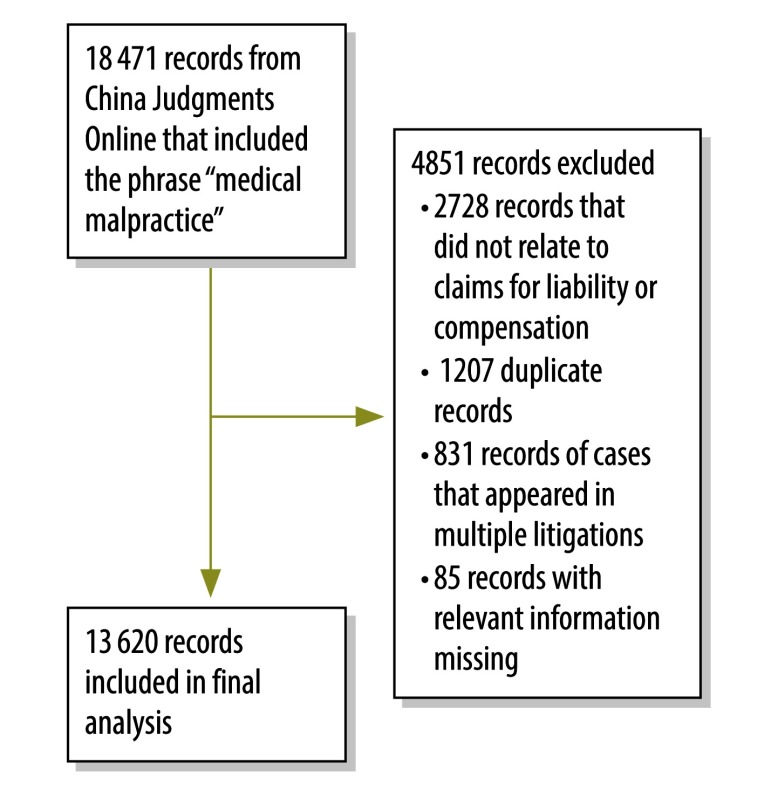
Flowchart of the selection of records of medical litigation, China, 2010–2015

We searched China Judgments Online for records – of litigation cases that ended between 1 January 2010 and 5 November 2015 – that included the phrase “medical malpractice”. We then excluded records that did not include compensation disputes or claims of medical malpractice liability, records from which any basic information was missing and duplicate records of individual cases (Fig. 1). If one case was reported in multiple records, we only kept the record of the final judgement.

### Data extraction and analysis

Using Python version 2.7.9 (Python Software Foundation, Beaverton, USA), we extracted relevant data – i.e. location of the court, the year the litigation ended, the medical specialization involved, the severity of any reported injury (Box 1), the type of allegation raised by the plaintiff – including any alleged shortcomings in the health care received – and the outcome of the litigation – from the selected records. We used the reported disease or injury to categorize each case into one of 14 medical specializations. We then used Stata version 13 (StataCorp. LP, College Station, USA) to produce summaries of the extracted data.

Box 1 **Categories of the severity of injury, China, 2002**

**Death**

**Severe disability**

- Level 1: for example, persistent vegetative state, quadriplegia

**Medium disability**

- Level 2: for example, blindness, loss of one kidney
- Level 3: for example, loss of one eye, loss of one lung
- Level 4: for example, loss of entire stomach, loss of bladder
- Level 5: for example, incontinence, loss of swallowing function

**Minor disability**

- Level 6: for example, loss of three quarters of stomach, loss of half of liver
- Level 7: for example, loss of two thirds of stomach, loss of third of liver
- Level 8: for example, loss of half of stomach, loss of quarter of liver
- Level 9: for example, loss of two thirds of an auricle
- Level 10: for example, thyroid hypofunction

Source: Chinese Ministry of Health.^27^

## Results

Of the 13 620 litigation records included in our analysis, about 65% (8893) resulted in a civil judgement and the rest in a civil ruling (Table 1). About half and a third of the records related to court proceedings in eastern and central China, respectively (Table 1). According to the data held in China Judgments Online, the annual numbers of cases of medical malpractice litigation increased substantially over our study period, from just 75 in 2010 to 6947 in 2014 (Table 1). Over 80% of the cases we investigated were represented by just four medical specializations: internal medicine, general surgery, orthopaedics and obstetrics and gynaecology (Table 1). Nearly all (5469; 89.6%) of the 6105 cases for which injury severity was recorded were either fatal cases or cases of minor injury (Table 1). Among the 5408 plaintiffs for whom allegations were recorded, the most frequently proposed allegation was a lack of consent or notification (1356; 25.1%), followed by misdiagnosis (1172; 21.7%), delay in treatment (1145; 21.2%) and alteration or forgery of medical records (975; 18.0%). For 11 014 cases, information on whether the plaintiff withdrew the lawsuit before a verdict, received compensation or did not receive compensation after a verdict was available. The records of the other cases focused on the administrative procedures of the trials, such as applications for retrial, rather than on decisions as to whether or not medical malpractice had occurred. Of the plaintiffs with known litigation outcomes, 7482 (67.9%) received monetary compensation, for instance to compensate for death or disability, emotional harm, funeral expenses and/or the living expenses of dependents, and 2121, or about 19%, withdrew their lawsuits (Table 1).

**Table 1:** Records of medical malpractice litigation, China, 2010–2015

Category	No. of records with relevant data (%)
**Legal category (*n* = 13 620)**	
Civil judgement	8893 (65.3)
Civil ruling	4727 (34.7)
**Region (*n* = 13 587)**^a^	
Central	4365 (32.1)
Eastern	6283 (46.2)
Western	2939 (21.6)
**Year litigation ended (*n* = 13 453)**	
2010	75 (0.6)
2011	97 (0.7)
2012	213 (1.6)
2013	1359 (10.1)
2014	6947 (51.6)
2015	4762^b^ (35.4)
**Primary claim (*n* = 5408)**	
Allergic reactions	115 (2.1)
Alteration or forgery of medical records	975 (18.0)
Delay in treatment	1145 (21.2)
Improper treatment	306 (5.7)
Lack of consent or notifications	1356 (25.1)
Lack of prevention	41 (0.8)
Lack of professional qualifications	59 (1.1)
Misdiagnosis	1172 (21.7)
Other adverse reactions	239 (4.4)
**Medical specialization (*n* = 8945)**	
Dermatology and venereology	136 (1.5)
Emergency	124 (1.4)
General surgery	1350 (15.1)
Infectious diseases	110 (1.2)
Internal medicine	3500 (39.1)
Obstetrics and gynaecology	1251 (14.0)
Oncology	296 (3.3)
Ophthalmology and otolaryngology	411 (4.6)
Orthopaedics	1283 (14.3)
Paediatrics	160 (1.8)
Plastic surgery	32 (0.4)
Psychiatric	138 (1.5)
Reproductive health	139 (1.6)
Traditional Chinese medicine	15 (0.2)
**Severity (*n* = 6105)**	
Death	4111 (67.3)
Medium injury	493 (8.1)
Minor injury	1358 (22.2)
Severe injury	143 (2.3)
**Outcome of litigation (*n* = 11 014)**	
Case withdrawn	2121 (19.3)
Compensation	7482 (67.9)
No compensation	1411 (12.8)

^a^ For our analyses, central China was represented by the provinces of Anhui, Heilongjiang, Henan, Hubei, Hunan, Jiangxi, Jilin and Shanxi, eastern China by the provinces of Beijing, Fujian, Guangdong, Hainan, Hebei, Jiangsu, Liaoning, Shandong, Shanghai, Tianjin and Zhejiang, and western China by the provinces of Chongqing, Gansu, Guizhou, Qinghai, Shaanxi, Sichuan and Yunnan and the Guangxi, Inner Mongolia, Ningxia, Tibet and Xinjiang autonomous regions. No corresponding data were available for Chinese Taipei or the Hong Kong Special Administrative Region or Macau Special Administrative Region.

^b^ Number recorded by 5 November.

Note: Inconsistencies arise in some values due to rounding.

Over our study period, the numbers of cases of medical malpractice litigation and the incidences of such litigation – both per million population and per 1000 physicians – generally appeared higher in eastern and central China than in western China (Table 2).

**Table 2:** Cases of medical malpractice litigation, China, 2010–2015

Area	No. of cases			
	Total	With compensation		
		Total	Per million population**^a^**	Per 1000 physicians**^a^**
**Provinces**				
Anhui, central China	842	422	7.1	5.0
Beijing, eastern China	721	477	23.6	6.8
Chongqing, western China	500	240	8.2	4.8
Fujian, eastern China	243	132	3.5	2.2
Gansu, western China	147	62	2.4	1.5
Guangdong, eastern China	652	315	3.0	1.9
Guizhou, western China	162	90	2.6	2.0
Hainan, eastern China	19	9	1.0	0.6
Hebei, eastern China	605	348	4.8	2.8
Heilongjiang, central China	113	63	1.6	0.2
Henan, central China	1578	839	8.9	5.4
Hubei, central China	554	269	4.7	2.8
Hunan, central China	577	322	4.9	2.8
Jiangsu, eastern China	1150	584	7.4	4.3
Jiangxi, central China	161	137	3.1	2.2
Jilin, central China	389	202	4.6	3.4
Liaoning, eastern China	522	254	9.2	1.4
Qinghai, western China	27	16	2.8	1.8
Shaanxi, western China	415	171	4.6	2.6
Shandong, eastern China	1131	660	6.9	3.5
Shanghai, eastern China	612	495	21.1	9.5
Shanxi, central China	151	82	2.3	1.0
Sichuan, western China	588	301	3.7	2.0
Tianjin, eastern China	171	91	6.7	3.0
Yunnan, western China	173	114	2.5	1.8
Zhejiang, eastern China	457	255	4.7	2.0
**Autonomous regions**				
Guangxi, western China	465	260	5.6	3.5
Inner Mongolia, western China	96	56	2.3	1.0
Ningxia, western China	88	47	7.4	3.8
Tibet, western China	3	2	0.7	0.5
Xinjiang, western China	275	152	6.5	2.9

^a^ For our analyses, the size of the population and number of physicians in each study area were assumed to be those recorded in 2011.^28^

## Discussion

The health system reform in China, launched in 2009, was designed to promote universal health coverage and has already led to some remarkable achievements. Between 2005 and 2011, for example, coverage of the national population with health insurance increased from less than 50% to about 95%.^29^ However, concerns have been raised that, at least in rural China, health-care reforms may be focused too much on cost containment and may pay too little attention to care quality.^30^ As mean per-capita incomes and insurance coverage increase in China, the population’s demand for health care – and particularly for high-quality medical services – also increases.

While there is no official system for the measurement of health-care quality in China,^31^ the mandatorily submitted records of medical malpractice litigation could form a nationally representative sample of medical malpractice. While most other indicators of health care – e.g. routine medical records – tend to focus on clinical signs and symptoms, litigation records provide information not only on treatment but also on adverse outcomes associated with mistakes or other shortcomings in health care. Analysis of the features of – and temporal trends in – malpractice litigation may also allow the development of more effective methods for improving health-care quality. In addition, the wider dissemination of data on malpractice litigation may reduce the future incidence of medical malpractice.

Our study indicates that, in China, medical malpractice litigation has become much more common than previously reported.^5^^,^^25^ However, we cannot determine if the increasing frequency of such litigation reflects a decrease in the general quality of health care. Between 2010 and 2015, similar increases in the annual numbers of cases of medical malpractice litigation have been observed in Japan,^32^ Mexico^33^ and the USA.^6^

The geographical differences that we observed – in the total number of medical malpractice cases, the number of cases per million population and the number of cases per 1000 physicians, can probably be partially explained in terms of the uneven distribution of health-care resources across China. The more developed eastern China tends to have more medical resources than central or western China.^34^ Eastern China also appears to have relatively high incidences of medical malpractice litigation per million population and per 1000 physicians – although we were unable to determine what proportions of the incidence recorded for a study area were represented by the residents of the study area and by people from other areas who had travelled for treatment. One aim of the health system reforms in China is to reinvigorate health-care services in the country’s less developed areas. Our study results may re-emphasize the importance of a more equal allocation of health-care resources across China.

Another possible factor in the uneven geographical distribution of medical malpractice litigation across China is geographical variation in access to – and/or attitudes towards – justice. In some areas of Central and Latin America, nongovernmental organizations have assisted plaintiffs in litigation for access to essential medicines.^35^ It seems possible that, in the more developed areas of China, patients or patients’ families may be more likely to be encouraged to file lawsuits by nongovernmental organizations or other forms of social assistance.

Internal medicine, general surgery, orthopaedics and obstetrics and gynaecology were found to be the leading specializations of malpractice litigations, which is consistent with the previous literature.^5^^,^^6^^,^^36^ The three most common allegations – misdiagnosis, delay in treatment and lack of consent or notifications – were all aspects of process quality.

Most of the litigation cases we investigated were associated with either the most severe or minor adverse outcomes. Severe adverse outcomes are probably those most likely to result in litigation while malpractice with minor adverse outcomes may be relatively common simply because most adverse outcomes are of minor severity. It has been suggested that medical error may be the third leading cause of death in the USA.^37^ In China, fatal medical errors require much more attention.

Of the cases we investigated, 68% ended with monetary compensation. This percentage is close to the corresponding values previously reported in China (67%), Japan (60%) and the USA (56%) but substantially higher than the value reported for Canada (33%).^5^^,^^6^^,^^32^^,^^38^ The between-country differences in this proportion probably reflect between-country differences in legal and medical systems and socioeconomic backgrounds.

Our study had several limitations. First, medical malpractice cases presumably represent only a small proportion of the patients who receive health care of poor quality. Second, we only analysed data from a single online database and it seems unlikely that this database held records for all of the cases of medical malpractice litigation that occurred in China over our study period. The proportion of such cases that were included in the database may also have changed during our study period. Third, in our analysis we ignored some variables that were recorded in the database, e.g. the amount of compensation awarded and the relative contributions made, to each lawsuit, by lawyers and insurers. Finally, if we are to use litigation records as an indicator of health-care quality, we probably have to assume that the judiciary system involved is fair, independent and strong and that the collection of data on medical malpractice litigation is reasonably comprehensive or, at least, nationally representative.

In conclusion, in the absence of more robust and traditional indicators, analysis of medical litigation records may give useful information on health-care quality. Medical malpractice is both a legal issue and a health system issue, since it involves governments, health providers, insurance companies, legal systems and patients. More studies are necessary on this topic, not only for studying health-care quality but also, ultimately, for strengthening health systems.
